# Association between Physical Activity Levels and Physiological Factors Underlying Mobility in Young, Middle-Aged and Older Individuals Living in a City District

**DOI:** 10.1371/journal.pone.0074227

**Published:** 2013-09-06

**Authors:** Luca Laudani, Giuseppe Vannozzi, Zimi Sawacha, Ugo della Croce, Andrea Cereatti, Andrea Macaluso

**Affiliations:** 1 Department of Human Movement, Social and Health Sciences, University of Rome Foro Italico, Rome, Italy; 2 Department of Information Engineering, University of Padua, Padua, Italy; 3 Information Engineering Unit, Department of POLCOMING, University of Sassari, Sassari, Italy; University of Valencia, Spain

## Abstract

Maintaining adequate levels of physical activity is known to preserve health status and functional independence as individuals grow older. However, the relationship between determinants of physical activity (volume and intensity) and physiological factors underlying mobility (cardio-respiratory fitness, neuromuscular function and functional abilities) is still unclear. The aim of this study was to investigate the association between objectively quantified physical activity and a spectrum of physiological factors underlying mobility in young, middle-aged and older individuals living in a city district. Experiments were carried out on 24 young (28±2 years), 24 middle-aged (48±2 years) and 24 older (70±3 years) gender-matched volunteers. Physical activity was monitored by a wearable activity monitor to quantify volume and intensity of overall physical activity and selected habitual activities over 24 hours. Ventilatory threshold was assessed during an incremental cycling test. Torque, muscle fiber conduction velocity and agonist-antagonist coactivation were measured during maximal voluntary contraction of knee extensors and flexors. Ground reaction forces were measured during sit-to-stand and counter-movement jump. *K-means* cluster analysis was used to classify the participants’ physical activity levels based on parameters of volume and intensity. Two clusters of physical activity volume (i.e., high and low volume) and three clusters of physical activity intensity (i.e. high, medium and low intensity) were identified in all participants. Cardio-respiratory fitness was associated with volume of overall physical activity as well as lying, sitting, standing, walking and stair climbing. On the other hand, neuromuscular function and functional abilities showed a significant association with intensity of overall physical activity as well as postural transition, walking and stair climbing. As a practical application, the relative role played by volume and intensity of overall physical activity and selected habitual activities should be taken into account in the design of preventative training interventions to preserve mobility as individuals grow older.

## Introduction

The World Health Organization recognizes physical inactivity as the fourth leading risk factor for global mortality [1]. Levels of physical activity (PA) have shown an unambiguous relationship with the incidence of numerous cardiovascular and neuromuscular diseases [2], [3], particularly across urban areas [4]. The highest percentage of sedentary individuals is older than 65 years, as levels of PA decrease significantly with age [5], [6]. Moreover, aging is accompanied *per se* by an inexorable decline of the major physiological factors underlying mobility, such as cardio-respiratory fitness and neuromuscular function, which are exacerbated by inactivity [7], [8], [9]. The age-related decline in cardio-respiratory fitness is determined primarily by morphological changes of the cardiovascular system [7]. As a result, older individuals show a reduced cardiac output and oxygen uptake during exercise [8]. Furthermore, changes of neural system and muscle fibers occurring with aging lead to a decline of neuromuscular function [9]. This is associated to a reduced ability to generate muscle strength and power and consequently carry out daily living activities [10], [11].

Epidemiological studies based on the use of questionnaires have shown that, in older adults, self-reported levels of PA are associated with independence in activities of daily living [12] and the number of disability-free years [13]. More recently, wearable inertial devices have been adopted to objectively assess PA levels in both young [14] and older individuals [15], [16]. Using such devices, Nokes et al. [17] have found that volume and intensity of PA were significant predictors of cardio-respiratory fitness, indexed by the 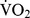
_max_ in middle-aged women. Another study has reported a significant association between objectively measured walking patterns of everyday life, and muscle strength and power of the lower extremities in older adults with functional limitations [18]. To the best of the authors’ knowledge, however, the relationship between levels of PA, described by volume and intensity, and a combined evaluation of the major determinants of mobility (e.g., cardio-respiratory fitness, neuromuscular function and functional abilities) has never been assessed jointly in one study across the age-spectrum from young to older city-dwelling individuals.

Techniques of data mining are often used to extract the essential and relevant information from large databases through the use of specific algorithms, in order to identify a set of meaningful patterns and relationships between the data [19]. One of the limitations of previous studies on the association between PA levels and mobility was that identifying levels of PA, i.e. distinguishing between individuals with low and high volume or intensity of PA, was carried out by establishing arbitrary *a-priori* cut-off points [20] or the median value [21] of a predetermined PA determinant (i.e. total count of activity). In contrast, cluster analysis [22], which is one of the most common data mining techniques, makes it possible to define PA levels by data-driven grouping and to include into the analysis all PA determinants.

The aim of this study was to look at the association between objectively quantified PA levels and a spectrum of physiological factors underlying mobility in young, middle-aged and older individuals living in a city district. It was hypothesized that the intensity of PA would have been more strongly related to factors underlying mobility than the volume of PA.

## Materials and Methods

### Ethics Statement

All participants gave their written informed consent according to the declaration of Helsinki. The experimental protocols were approved by the Ethics Committee of the University of Rome La Sapienza.

### Participants

Seventy-two volunteers were recruited from the metropolitan area through local advertisement. Participants were assigned to three age groups, each composed by 24 individuals (12 males and 12 females) of different age: one group of young individuals (age: 28±2 years); one group of middle-aged individuals (age: 48±2 years); one group of older individuals (age: 70±3 years). Older subjects were selected according to the exclusion criteria to define “medically stable” older individuals for exercise studies, as proposed by Greig et al [23]. Each participant filled the International Physical Activity Questionnaires long form, which provides a comprehensive evaluation of daily PA habits and has been validated for use in urban context [24]. Only sedentary individuals who were not engaged in regular training or sport practice more than 3 times a week, for more than 40–60 minutes each time, were included in the study.

### Physical Activity Monitoring

Participants’ habitual PA was monitored under free living conditions by means of a portable activity monitor (IDEEA, MiniSunLLC, USA; 32-Hz sampling frequency) that integrates inclinometers and two-component accelerometers conveniently located on the participant’s body. A total of five inertial sensors were placed: one on the chest just below the sternal angle; two on the frontal part of thighs along the median axis; and two under the feet at the plantar arc level. All participants wore the activity monitor in the early morning of a weekday and removed it after 24 hours. Data were downloaded to a personal computer and analyzed off-line (MiniSun GaitView 2 2.2). The outputs provided were represented by specific type, duration (i.e., volume) and speed (i.e., intensity) of daily living activities including 4 locomotor tasks (walking, running, stair climbing and ascending), 3 body postures (sitting, lying, and standing), and 3 postural transitions (lying to sitting, lying to standing, sitting to standing). Identification accuracy of such activities by the activity monitor has been shown to range from 98.2 to 99.5% [25]. The overall volume of PA was quantified by the total count of all daily living activities recognized over the 24 hours, whilst the overall intensity of PA was quantified by the average speed of all activities. Pooled correlations between predicted and actual speeds have been shown to be high (*r* = 0.98) [25].

### Mobility Assessment

Evaluation of major physiological determinants of mobility involved the participants to attend the laboratory in two different occasions, separated by at least one week.

#### First laboratory session

In this session, subjects’ anthropometric characteristics and cardio-respiratory fitness were evaluated. Initially, volunteers were fully familiarized with all experimental procedures. Stature and body mass were then measured for the anthropometric assessment.

*Cardio-respiratory fitness:* A portable metabolimeter (K4b^2^, COSMED, Italy) was used to measure the oxygen uptake (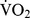) and carbon dioxide production (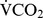). Heart rate (HR) was recorded by means of a portable heart rate monitor (Polar, Finland) whose output was telemetrically transmitted and recorded in the K4 system. Each individual wearing the K4 system performed an incremental exercise test on a bicycle ergometer (Monark 824E, Sweden), with workload increments of 10 W per minute according to Wasserman et al. [26]. The ventilatory threshold (T*vent*) was then determined by using the ventilatory equivalent method, i.e., a systematic increase in the ventilatory equivalent of O_2_ (the ratio between pulmonary ventilation and 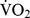), with no concomitant rise in the ventilatory equivalent of CO_2_ (the ratio between pulmonary ventilation and 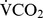) and by using the V-slope method of Beaver et al. [27]. The V-slope method involves the analysis of 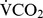 as a function of 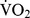 and assumes that the threshold corresponds to the break in the linear 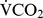-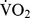 relationship. The final value of T*vent* was calculated as the average of the two values obtained with the two methods. Workload, HR and 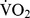 corresponding to T*vent* were then used for further analysis, as previously proposed [28], [29], [30].

#### Second laboratory session

In this session, subjects were assessed for functional abilities and neuromuscular function (i.e. muscle strength and neural activation).

*Functional abilities:* First, participants performed a sit-to-stand task (STS) on a six-component dynamometric platform (BERTEC, 4080-10, Columbus, Ohio, USA; 100 Hz sampling frequency). The height of the chair was adjusted to the participant’s shank length in order to obtain a 90-degree angle at the knee joints in all individuals [31]. Each subject performed 3 trials at their fastest speed, with 1-min rest in between. All the subjects were then evaluated on the force platform by performing a vertical countermovement jump on both feet (CMJ). Each subject started from an upright posture and the jump was preceded by a rapid counter-movement. The subjects kept their hands on their hips throughout each jump to avoid interference due to any arms’ movement. Each jump was performed at least three times, with 3-min rest in between. Ground reaction forces during both STS and CMJ tasks were filtered using a digital, low-pass, second-order, Butterworth filter (cut-off frequency set at 15 Hz). The force signal measured by the force platform was then analyzed, as has been proposed by Davies and Rennie [32], to obtain the vertical velocity v(t) of displacement of the body centre of gravity as follows:
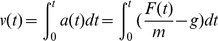
where *a* is the vertical acceleration imposed by muscle contraction to the body centre of gravity, *F* is the vertical force measured by the platform, *m* is the body mass of the subject and *g* the acceleration of gravity. Vertical (V) power was calculated by multiplying the vertical force by the vertical velocity. The values of vertical force and vertical velocity corresponding to peak power were considered for this study and named optimal force (F_opt_) and optimal velocity (v_opt_), respectively [33]. During the STS movement, also the antero-posterior (AP) component of peak force and power was calculated. For both STS and CMJ the best of the 3 trials, i.e. the one with the highest V-peak power, was selected for further analysis.

*Muscle strength:* Participants were tested for isometric maximal voluntary contraction (MVC) of knee extensor and flexor muscles on the dominant lower limb by using a dynamometer (Kin Com, Chattanooga, TN). Participants warmed up on an exercise bicycle for 5 min at a low resistance before performing the strength test. During the test, participants were seated comfortably in the dynamometer chair, with their trunk erect and fastened by three crossing belts. They were positioned so that a 90° angle at the knee joint was obtained. The MVC task consisted of a quick increase to a maximum in the force exerted by the leg. A target line was always set on the computer screen at a value 20% higher than the best performance [34]. Participants were able to follow their performance on the computer screen and were verbally encouraged to achieve a maximum and to maintain it for at least 2 s before relaxing. MVC was calculated as the largest 1-s average reached within any single force recording. A minimum of 3 attempts was performed separated by 3 min, and that with the highest force value was chosen as MVC. Participants were asked to make a further attempt if the MVC of their last trial exceeded that of previous trials. Rate of force development (RFD) was also calculated during both knee extension and flexion MVC tasks.

*Neural activation:* Throughout strength testing, surface electromyography (sEMG) from the *vastus lateralis* (VL) and *biceps femoris* (BF) muscles of the dominant limb was recorded by means of a linear array of four electrodes (silver bars 5 mm long, 1 mm diameter, 10 mm apart; LISiN, Turin, Italy). First, electrode position was identified between the motor point and the distal tendon, in a direction parallel to the muscle fibers, after checking on the computer screen that there was clear propagation in one direction of the action potentials without change in shape [35]. Second, after gentle skin abrasion with abrasive paste (Meditec-Every, Parma, Italy), the electrodes were attached on the skin. A ground electrode was placed around the ankle of the contra lateral limb. sEMG signals were detected in a single differential mode and the double differentials were computed off-line. The signal was preamplified (×1000), amplified (×1 for BF and ×2 for the VL), band-pass filtered (5 Hz–1 kHz) and sampled at 2048 Hz.

All data collected during the experiments were analyzed off-line (LabView 8.0, National Instruments, Texas). The sEMG variables of interest were the root mean square (RMS) and muscle fiber conduction velocity (MFCV). According to Merletti et al. [35], RMS was computed from the central single differential signal over non-overlapped adjacent epochs of 500 ms. The same windowing was used to estimate the average MFCV from the two double differentials by means of the EMG cross-correlation function technique. Estimates of MFCV were accepted only when cross-correlation values were higher than 0.7, which has been reported to be an acceptable correlation coefficient for VL, due to the fibers’ orientation [36].

Agonist-antagonist coactivation was calculated during the knee extension as previously described in Macaluso et al. [34]. Briefly, the RMS data recorded from *biceps femoris* muscle during the knee extension were divided by the corresponding RMS recorded during the knee flexion MVC and expressed as a percentage. The RMS data recorded from *vastus lateralis* muscle during the knee flexion were divided by the corresponding RMS recorded during the knee extension MVC and expressed as a percentage.

### Statistical Analysis

Multivariate analysis of variance (MANOVA) was performed on all quantitative variables of PA and mobility and paired-sample t-test was used to identify significant differences between age groups. A data mining approach was used to automatically extract information hidden in the data [19]. Specifically, cluster analysis was performed separately for volume and intensity of PA parameters in order to identify any natural grouping that may exist in the involved sample of individuals, with the advantage of taking into account several parameters rather than a single one for each individual [22]. The clustering function *K-means* (Matlab software, R2008b) was used, and the standard euclidean distance was chosen in forming the clusters. *K-means* uses an iterative algorithm that minimizes the sum of distances from each object to its cluster centroid over all clusters and moves objects between clusters until the sum cannot be decreased further. The number of cluster is increased at each solution until an empty cluster is created and the solution that generates clusters that are better separated than previous solutions is chosen. As a result, the obtained clusters represented individuals characterized by different levels of either volume or of intensity of PA. The clusters for either volume or intensity of PA were then used as independent variables in a multivariate one-way analysis of covariance (MANCOVA), in which all quantitative variables of PA and mobility were the dependent variables and age the covariate. Follow-up tests were conducted to evaluate pair-wise differences between clusters, using a Bonferroni-Holm correction when appropriate. Pearson product-moment correlation coefficients between pairs of quantitative variables were calculated across all participants to evaluate the relationship between PA and mobility in all participants. MANOVA, MANCOVA and correlation analyses were performed using SPSS software (version 20.0, SPSS, Inc., Chicago, IL). A significance level of P<0.05 was adopted.

## Results

Descriptive statistics of PA data in young, middle-aged and older participants are reported in Table 1. Mean speed of walking and stair climbing were significantly lower in older than in both young and middle-aged participants. Duration of stair climbing and sitting was significantly higher in middle-aged than in older participants. There were no significant differences between age groups in overall activity, both in terms of total count and average speed. As shown in table 2, most parameters of mobility were significantly lower in older than in both young and middle-aged participants, while there were no differences between age groups in parameters of neural activation.

**Table 1:** Representative parameters of objectively measured physical activity for each age group.

	Y	M	O
	(n = 24)	(n = 24)	(n = 24)
**Lying**			
Total time (min)	415.5±110.8	381.1±86.4	448.0±103.0
**Sitting**			
Total time (min)	622.5±92.6	658.9±163.3†	546.6±108.4
**Standing**			
Total time (min)	297.9±93.5	307.3±120.4	341.3±118.6
**Postural transition**s			
Total time (min)	7.6±4.0	9.4±8.1	6.7±3.7
Mean speed (m min^−1^)	0.41±0.20	0.48±0.26	0.43±0.41
**Walking**			
Total time (min)	81.9±41.2	68.0±30.4	82.9±38.2
Mean speed (m min^−1^)	69.6±7.5*	68.5±7.3†	60.0±6.3
**Stair climbing**			
Total time (min)	5.3±2.8*	3.8±1.9	3.2±2.8
Mean speed (m min^−1^)	77.2±7.2*	75.5±7.5†	67.0±7.4
**Overall activity**			
Total count	21525.4±5834.4	20487.3±5909.5	22576.8±5538.5
Mean speed (m min^−1^)	4.4±2.2	3.5±1.4	3.8±1.7

Values are means ± SD of parameters relative to 24 h instrumental monitoring of habitual physical activity.

* = significantly different between young (Y) and older (O) participants;

† = significantly different between middle-aged (M) and older participants.

**Table 2:** Representative parameters of physiological factors underlying mobility for each age group.

	Y	M	O
	(n = 24)	(n = 24)	(n = 24)
**T** ***vent***			
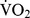 (ml kg^–1^ min^–1^)	27.0±5.0* ‡	22.7±6.1†	16.0±2.8
HR (beats min^–1^)	141.4±14.0*	143.2±13.1†	116.0±14.0
Load (W)	130.8±25.7*	126.2±42.0†	74.7±17.8
**MVC**			
Peak torque (N m kg^–1^)	2.46±0.68* ‡	1.91±0.58	1.55±0.47
RFD (N m s^–1^)	1095.0±328.2*	1007.9±311.5	855.4±270.3
**CMJ**			
Flight time (s)	0.47±0.06* ‡	0.38±0.07†	0.30±0.06
V-peak power (W kg^–1^)	52.0±14.7*	47.9±22.2†	24.7±5.9
**STS**			
V-peak power (W kg^–1^)	16.4±4.2* ‡	12.7±3.4	10.5±3.7
AP-peak power (W kg^–1^)	1.55±0.65*	1.20±0.53	1.02±0.43
**sEMG**			
MFCV (m s^−1^)	4.43±0.47	4.06±0.67	4.31±0.57
Coactivation (%)	29.6±20.5	31.7±22.5	26.2±22.8

Values are means ± SD of the following mobility parameters: oxygen consumption (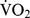), heart rate (HR), and mechanical load at ventilatory threshold (T*vent*) during incremental cycling; vertical (V) and antero-posterior (AP) peak power during chair rising (STS) at maximal intensity; flight time and peak power during a countermovement jump (CMJ); peak torque and rate of force development (RFD) during isometric maximum voluntary contraction (MVC) of knee extensor muscles; muscle fiber conduction velocity (MFCV) of *vastus lateralis* and the antagonist coactivation from surface electromyography (sEMG) during knee extension maximal voluntary contraction.

* = significantly different between young (Y) and older (O) participants;

† = significantly different between middle-aged (M) and older participants;

‡ significantly different between young and middle-aged participants.

### Association between PA Volume and Mobility

Cluster analysis on PA volume in all subjects led to definition of 2 distinct clusters: one cluster (n: 39) was characterized by high volume of PA (HPAv) and included 13 young, 13 middle-aged and 13 older participants, while the other cluster (n: 33) was characterized by low volume of PA (LPAv) and included 11 young, 11 middle-aged and 11 older participants. MANCOVA showed that individuals in the HPAv cluster had a significantly higher total activity count than individuals of the LPAv cluster (25827.6±3429.6 vs 16450.6±3188.7; *F* (1, 69) = 142.9, *p*<0.001). Time spent lying was significantly lower in HPAv (370.6±82.1 min) than LPAv (467.3±101.3 min; *F* (1, 69) = 20.0, *p*<0.001) and, consistently, HPAv individuals spent significantly less time sitting with respect to LPAv individuals (569.6±120.4 min vs 656.4±131.4 min; *F* (1, 69) = 8.9, *p*<0.01). Nevertheless, individuals of the HPAv cluster spent a significantly higher amount of time than individuals of the LPAv cluster during standing (376.0±108.3 min vs 243.9±62.7 min; *F* (1, 69) = 38.9, *p*<0.001), walking (98.0±36.2 min vs 53.5±19.4 min; *F* (1, 69) = 39.2, *p*<0.001), and stair climbing (4.8±3.0 min vs 3.3±2.0 min; *F* (1,70) = 6.0, *p*<0.05).

The statistical analysis of the differences in laboratory based physiological determinants of mobility between the 2 clusters showed that the 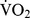 at T*vent* was significantly higher in participants of HPAv than in participants of LPAv (23.3±7.3 ml kg^–1^ min^–1^ vs 20.2±5.3 ml kg^–1^ min^–1^; *F* (1, 67) = 5.4, *p*<0.05). Consistently, correlation analysis in all subjects revealed a significant relationship between the time spent climbing stairs and 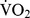 at T*vent* (r = 0.39; *p*<0.01).

### Association between PA Intensity and Mobility

Cluster analysis on PA intensity in all subjects led to definition of 3 distinct clusters: one cluster (n: 19) was characterized by high intensity of PA (HPAi) and included 9 young, 8 middle-aged and 2 older participants; one cluster (n: 32) was characterized by medium intensity of PA (MPAi) and included 13 young, 13 middle-aged and 8 older participants; and another cluster (n: 21) was characterized by low intensity (LPAi) and included 2 young, 5 middle-aged and 14 older participants. MANCOVA showed statistically significant differences between groups for the following parameters of PA intensity: overall average speed (*F* = 3.6; *p*<0.05), mean speed of postural transitions (*F* = 4.0; *p*<0.05), mean speed in walking (*F* = 30.5; *p*<0.001), and mean speed in stair climbing (*F* = 92.7; *p*<0.001). Individuals of the HPAi cluster showed significantly higher overall average speed than individuals of the MPAi cluster (4.9±2.2 m min^−1^ vs 3.6±1.3 m min^−1^; *p*<0.05). Consistently, mean speed of walking and stair climbing was significantly higher in HPAi (75.3±7.4 m min^−1^ and 83.7±5.1 m min^−1^, respectively) than MPAi (65.2±4.4 m min^−1^ and 73.3±2.9 m min^−1^, respectively) which, in turn, was significantly higher than LPAi (58.9±5.1 m min^−1^ and 63.5±4.4 m min^−1^, respectively). Mean speed of postural transitions was significantly higher in HPAi than LPAi (0.5±0.4 m min^−1^ vs 0.3±0.1 m min^−1^; *p*<0.05).

**Figure 1:**
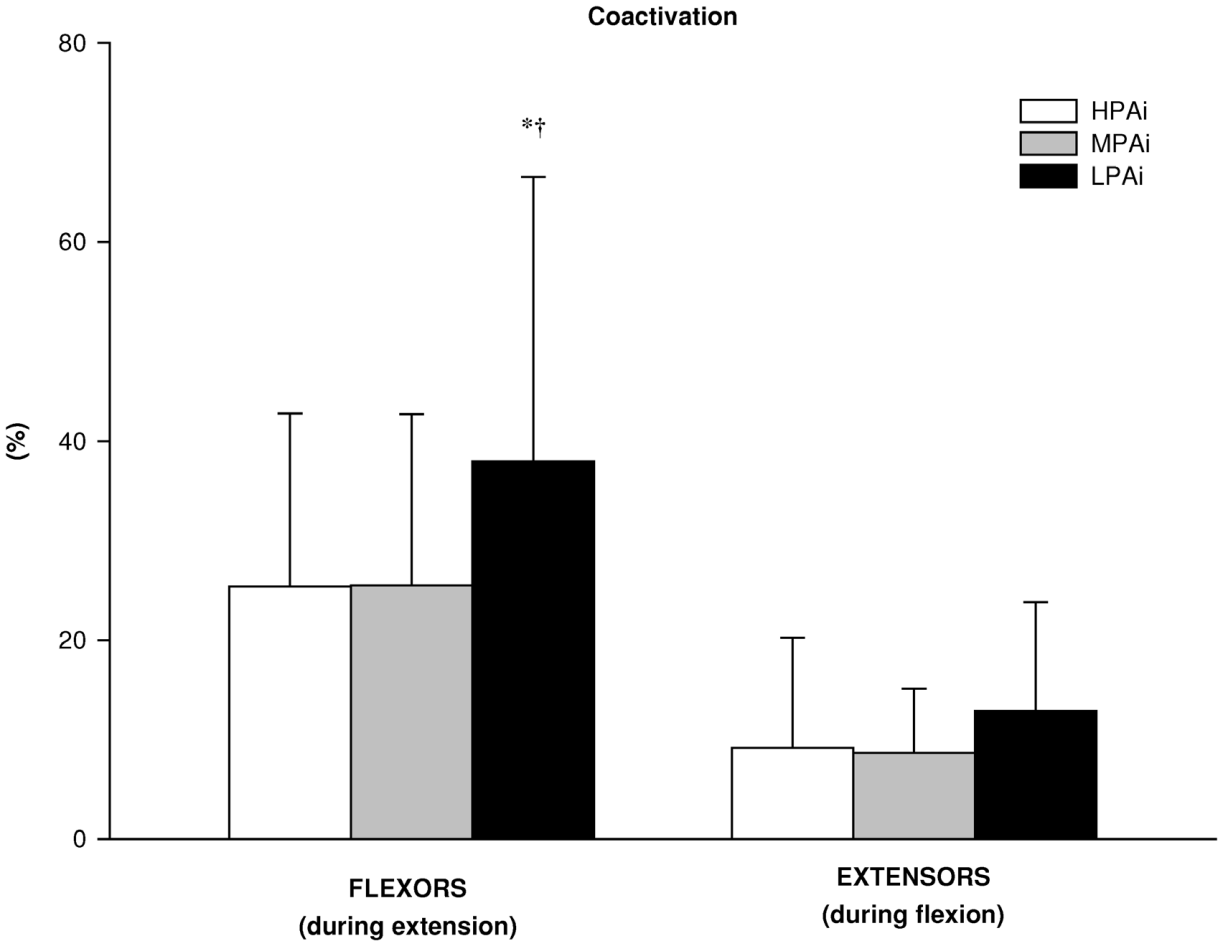
Neural activation in the 3 PA intensity clusters. Percentage coactivation of the *biceps femoris* muscle during the knee extension maximal voluntary contraction (MVC) and of the *vastus lateralis* muscle during knee flexion MVC in the high (HPAi; n: 19), medium (MPAi; n: 32) and low (LPAi; n: 21) PA intensity clusters. Data are expressed as means ± SD. *significantly different from MPAi; ^†^significantly different from HPAi.

**Figure 2:**
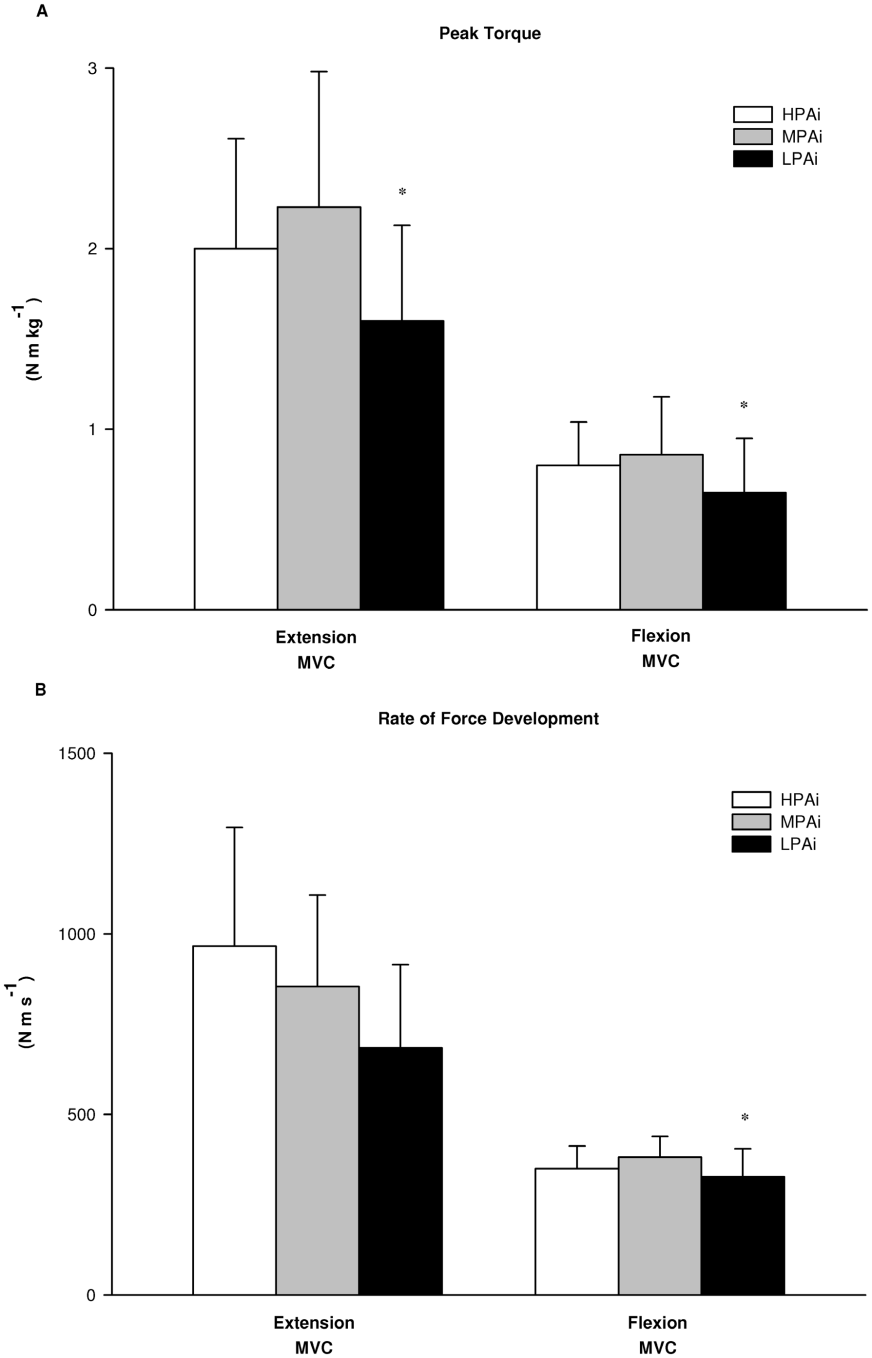
Muscle strength in the 3 PA intensity clusters. (A) Peak torque and (B) rate of force development of the knee extensor and flexor muscles during maximal voluntary isometric contraction (MVC) in the high (HPAi; n: 19), medium (MPAi; n: 32) and low (LPAi; n: 21) PA intensity clusters. Data are expressed as means ± SD. *significantly different from MPAi.

**Figure 3:**
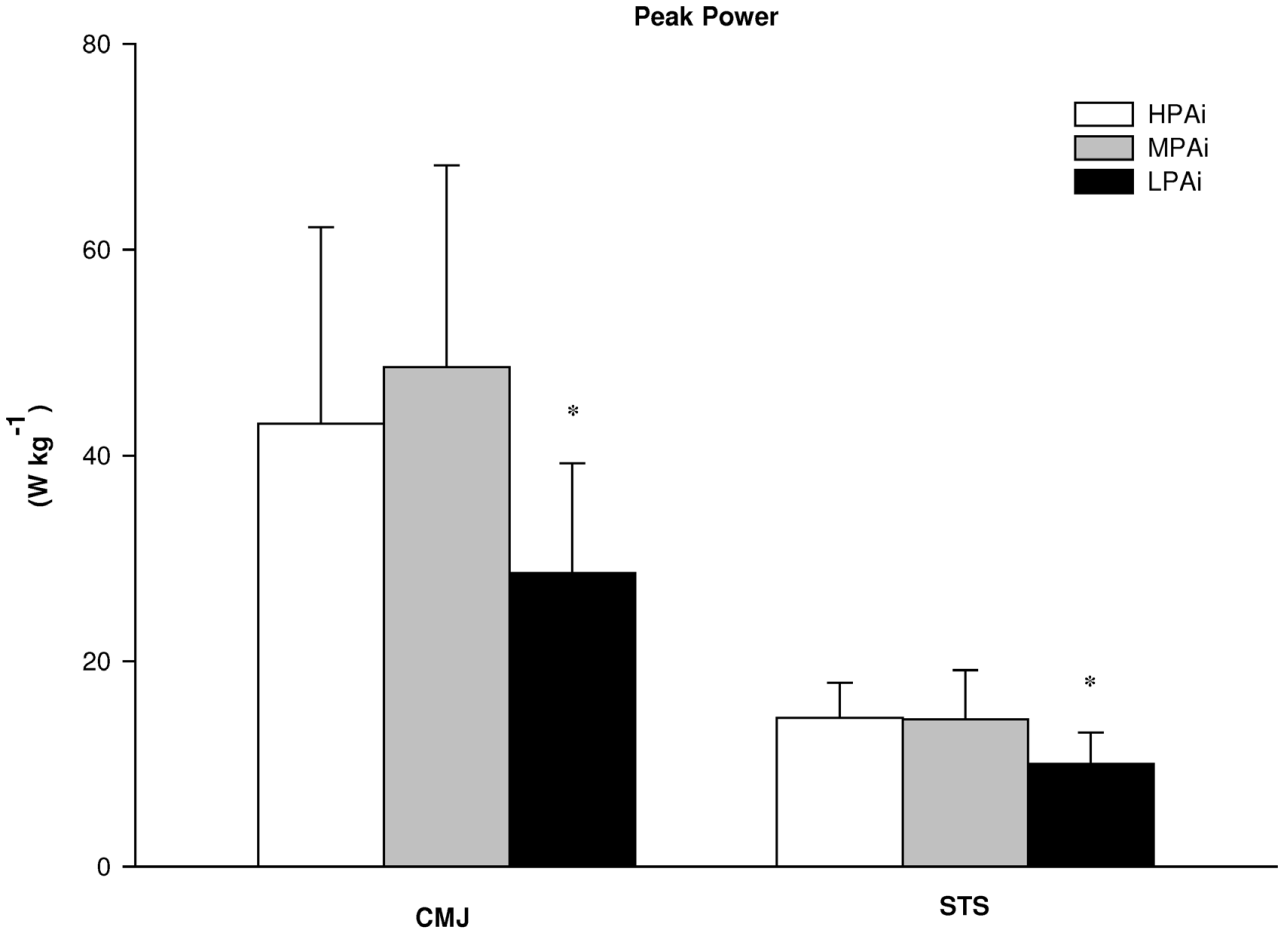
Functional abilities in the 3 PA intensity clusters. Vertical peak power during maximal counter-movement jump (CMJ) and sit to stand (STS) at maximal intensity in the high (HPAi; n: 19), medium (MPAi; n: 32) and low (LPAi; n: 21) PA intensity clusters. Data are expressed as means ± SD. *significantly different from MPAi.

The statistical analysis of the differences in laboratory based physiological determinants of mobility between clusters showed a significant main effect for the group factor (HPAi, MPAi and LPAi) on antagonist coactivation during knee extension MVC (*F* = 4.4; *p*<0.05), RFD during knee flexion MVC (*F* = 4.3; *p*<0.05), flight time (F = 3.2; *p*<0.05) and V-peak power (F = 3.7; *p*<0.05) during CMJ, V-peak power during STS (F = 3.9; *p*<0.05). There was a tendency toward significance for RFD (*F* = 2.8; *p* = 0.06) and peak torque (*F* = 2.4; *p* = 0.09) during knee extension MVC. As shown in Figure 1, follow-up tests revealed that antagonist coactivation was significantly lower in HPAi than in MPAi (*p*<0.05) and LPAi (*p*<0.05). Although there were no between-groups differences in peak torque and RFD during knee extension MVC and in peak torque during knee flexion MVC, RFD during knee flexion was significantly lower in LPAi than in MPAi (*p*<0.05) (Figure 2). Figure 3 shows V-peak power of both CMJ and STS, which were significantly lower in LPAi than in MPAi (*p*<0.05). There were no significant differences in measurements of MFCV and cardio-respiratory fitness between groups.

Correlation analysis in all subjects revealed a lack of association between the average speed of all daily living activities pooled together with measurements of neuromuscular function and functional abilities. In contrast, mean speed of single daily living activities was correlated with most of the laboratory based physiological determinants of mobility. For instance, mean speed of stair climbing showed a significant correlation with: flight time (*r = *0.45; *p*<0.01), V-peak power (*r* = 0.27; *p*<0.05) of CMJ; peak torque (*r* = 0.31; *p*<0.01) and RFD (*r* = 0.28; *p*<0.05) during knee extension MVC and peak torque (*r* = 0.28; *p*<0.05) during knee flexion MVC; AP-peak power (*r* = 0.35; *p*<0.01) and V-peak power (*r* = 0.41; *p*<0.01) during STS. Similarly, mean speed of walking was significantly correlated to: flight time (*r = *0.42; *p*<0.01), V-peak power (*r* = 0.29; *p*<0.05) of CMJ; peak torque (*r* = 0.26; *p*<0.05) and RFD (*r* = 0.26; *p*<0.05) during knee extension MVC and peak torque (*r* = 0.29; *p*<0.05) during knee flexion MVC; and AP-peak power (*r* = 0.24; *p*<0.05) and V-peak power (*r* = 0.34; *p*<0.01) during STS. The intensity of postural transitions was not correlated with any laboratory based physiological determinants of mobility.

## Discussion

To the best of authors’ knowledge, this is the first time that major physiological determinants of human mobility (cardio-respiratory fitness, neuromuscular function and functional abilities) have been assessed jointly in one study and compared to objectively measured PA levels in three age groups of individuals (young, middle-aged and older). Cluster analysis was used to classify the PA levels of participants based on either volume or intensity of both overall PA and selected habitual activities. The main finding is that in all participants volume of PA was associated to cardio-respiratory fitness, while intensity of PA was associated to neuromuscular function and functional abilities.

A lower intensity of daily living activities, such as walking and stair climbing, in older participants than in young and middle-aged participants is consistent with the finding of others studying similar age groups with either questionnaires [37] or PA monitors [18], [21], [15]. Similarly, descriptive statistics of physiological factors underlying mobility in the 3 groups of young, middle-aged and older individuals, as shown in Table 2, are consistent with the numerous laboratory studies carried out in the past years on cardio-respiratory fitness [7], [8], neuromuscular function [3], [38] and functional abilities [10], [39].

Levels of PA were defined in all participants based on either volume or intensity by means of a clustering approach [22], which has the advantage of including into the analysis all the PA determinants assessed in this study, i.e. locomotor tasks (walking, and stair climbing), body postures (lying, sitting, and standing) and postural transitions (lying to sitting, lying to standing, sitting to standing), rather than one PA determinant at a time. Feeding all these parameters into such a heuristic model that identifies the optimal grouping, i.e. low and high PA clusters, is a major strength of this study compared to establishing an arbitrary *a-priori* cut-off point [20] or the median value [21] of a predetermined PA determinant (i.e. total count). In other words, the adopted *k-means* cluster analysis is an unsupervised technique, which makes it possible to identify data-driven grouping by selecting the most relevant subset of PA parameters among those available in the database. As a result, total count and time spent lying, sitting, walking and stair climbing were identified as the most relevant subset of parameters related to PA volume; whereas mean speed of overall activity, walking and stair climbing levels were identified as the most relevant subset of parameters related to PA intensity.

Cardio-respiratory fitness was significantly associated to volume, but not to intensity, of overall PA and selected habitual activities, such as standing, walking and stair climbing. This is in agreement with Oja [40], who found evidence for a graded dose-response between the total volume of weekly PA and cardio-respiratory fitness. After all, the American College of Sports Medicine recommendations emphasize the importance of the total volume of weekly PA to preserve cardio-respiratory fitness while aging [41]. It is interesting to note that it was only when accounting for age, by using it as a covariate in the MANCOVA, that there was no association between PA intensity and cardio-respiratory fitness. By removing the covariate age, PA intensity and cardio-respiratory fitness would have been associated, with most of the individuals with low PA intensity and cardio-respiratory fitness being older and most of the individuals with high PA intensity and cardio-respiratory fitness being young.

Differences in neuromuscular function and functional abilities between the low- and high-PA volume groups were negligible, which is in contrast with the results of Fogelholm et al. [37], who estimated PA volume through questionnaires, but in line with Morie et al. [21] who measured PA volume through tri-axial accelerometers, as in our study. Such a discrepancy may likely be attributed to the inaccuracy of questionnaires to quantify PA volume with respect to accelerometers. Our data clearly show that both neuromuscular function (antagonist coactivation and RFD during MVC) and functional abilities (peak power during both STS and CMJ) were associated exclusively with intensity of overall PA and selected habitual activities, such as postural transitions, walking and stair climbing. Individuals of LPAi exhibited the lowest scores of neuromuscular function and functional abilities, thus indicating that they may be at higher risk of disability, regardless of age, than the remaining two groups. Our data are consistent with those of Puthoff et al. [18], who found that muscle strength and power of the lower extremities were not correlated with walking distance (volume) but with walking speed (intensity) in older adults with functional limitations. Although our results cannot prove a cause-effect relationship between PA and muscle strength and power of the lower extremities, PA is an independent factor that could potentially be controlled or modified in ordinary life, in a similar way to smoking and nutritional habits. Current guidelines recommend about 30 minutes of structured moderate intensity exercise 4 or 5 times per week for adults [41]. Our results suggest that carrying out habitual activities at high intensity, such as walking and stair climbing, may provide an adequate stimulus to overcome the age-related decline in neuromuscular function and functional abilities.

In conclusion, results of the present study pointed towards a significant association between PA levels and physiological determinants of mobility in young, middle-aged and older individuals living in a city district, with significant differences in the relative role played by volume and intensity of overall PA and selected habitual activities. While cardio-respiratory fitness was associated to PA volume, neuromuscular function and functional abilities showed a significant association exclusively with PA intensity. As a practical application, these findings should be taken into account in the design of preventative training interventions to improve physiological determinants of human mobility.
